# The efficacy and safety of moxibustion for chemotherapy-induced gastrointestinal adverse reaction: A protocol for systematic review and meta-analysis

**DOI:** 10.1097/MD.0000000000022042

**Published:** 2020-08-28

**Authors:** Han-Xiao Zhang, Cheng-Shun Zhang, Xiao-Qin Dai, Chuan-Yi Zuo, Peng Lv, Rui-Zhen Huang, Qian-Ning Mo, Yi-Feng Bai, Yi Zhou

**Affiliations:** a Acupuncture and Tuina School, Chengdu University of Traditional Chinese Medicine, 37 Shi-er-qiao Road, Jinniu District; b Department of Traditional Chinese Medicine, Sichuan Academy of Medical Sciences and Sichuan Provincial People's Hospital; c Hospital of Chengdu University of Traditional Chinese Medicine, 39 Shi-er-qiao Road, Jinniu District; d Medical Administration Department, Sichuan Academy of Medical Sciences and Sichuan Provincial People's Hospital; e Oncology Department, Sichuan Academy of Medical Sciences and Sichuan Provincial People's Hospital; f Department of Breast Surgery, Sichuan Academy of Medical Sciences and Sichuan Provincial People's Hospital, Chengdu, Sichuan Province, China.

**Keywords:** chemotherapy, gastrointestinal adverse reaction, meta-analysis, moxibustion, protocol, systematic review

## Abstract

**Background:**

Many cancer patients experience gastrointestinal adverse reaction during chemotherapy. Pharmacological interventions are commonly used to treat chemotherapy-induced gastrointestinal side effects but have various limitations. Clinical trials have indicated that moxibustion may alleviate gastrointestinal dysfunction and improve quality of life (QoL) after chemotherapy. This study aims to assess the efficacy and safety of moxibustion for chemotherapy-induced gastrointestinal adverse reaction through a systematic review and meta-analysis.

**Methods:**

All randomized controlled trials (RCTs) related to moxibution targeting chemotherapy-induced gastrointestinal adverse reaction will be searched in online databases, such as PubMed, EMBASE, the Cochrane Library, Web of Science, China National Knowledge Infrastructure (CNKI), the Chinese Scientific Journal Database (VIP Database) and WanFang Database from their inception to May 1, 2020. The primary outcome is the incidence and severity of chemotherapy-related gastrointestinal toxicities (nausea and vomiting, diarrhea and constipation). The secondary outcomes include the quality of life, biological parameters’ alteration, and adverse events. Study selection, data extraction, and assessment of risk of bias will be performed independently by 2 researchers. The Cochrane Collaboration's Review Manager (RevMan 5.3) software will be used to conduct the direct meta-analysis.

**Results:**

This study will provide a comprehensive review of the available evidence for the treatment of chemotherapy-induced gastrointestinal adverse reaction with moxibustion.

**Conclusion:**

The conclusion of this study will provide evidence to judge whether moxibustion is an effective and safety therapeutic intervention for chemotherapy-induced gastrointestinal adverse reaction.

**PROSPERO registration number:**

CRD42020182990.

## 1 Introduction

Cancer is a severe health problem that ranks to the leading or second cause of death worldwide.^[1,2]^ According to the GLOBOCAN Cancer Statistics, in 2018, 18.1 million population developed cancer, and 9.6 million people died from cancer worldwide.^[1]^

Chemotherapy is recognized an effective way during the anti-cancer treatment, while it has greatly improved overall survival in variety types of cancers, also brings side effects, such as chemotherapy-induced nausea and vomiting (CINV),^[3,4]^ chemotherapy-induced diarrhea (CID),^[5,6]^ chemotherapy-induced constipation (CIC),^[5]^ pain,^[7]^ and fatigue,^[8]^ which were greatly interfering the treatment course. Among these toxic side events, chemotherapy-induced gastrointestinal reactions, including CINV, CID and CIC, were the most widely influential and severe symptoms.

Current managements against those chemotherapy-related gastrointestinal adverse reactions were mainly pharmacological approaches, the drugs mainly focus on diminishing the severity of symptoms,^[4,5]^ may lead to extra adverse events. For instance, 5-HT_3_ receptor antagonists, the commonly used medications in treating CINV, are seemed to be associated with electrocardiogram alteration and increased risk of arrhythmia.^[9]^ Loperamide and octreotide, 2 mainly drugs during the treatment against CID, will lead to severe constipation, abdominal pain or dizziness.^[5,10]^ The drugs used in treating CIC are frequently related to increased risk of electrolyte imbalance, serious dehydration, and laxative dependence.^[11]^ Given that the commonly used pharmacological interventions have obvious side effects, and also heavy financial burden, a complementary and alternative therapy is needed, which take minimum adverse reaction, less economic burden and effectiveness into account.

Moxibustion, as a noninvasive external method in traditional Chinese therapy,^[12]^ is performed by burning herbal preparations containing dry Artemisia vulgaris on or above the skin at acupoints,^[13]^ to obtain the therapeutic effects in clinical practice. Traditional moxibustion techniques involve either direct moxibustion with a moxa cone or a moxa stick, whereas indirect moxibustion, achieved by placing certain types of materials such as salt, ginger or garlic between the skin and a burning moxa at the acupoint.^[14]^ According to Chinese Medicine theory, disease is the result of the body's disharmony, and moxibustion aims to counterbalance the disharmony, by strengthening the host immunity,^[15]^ strengthening the body resistance and eliminating pathogens from the body. A systematic review revealed that moxibustion is effective in alleviating chemotherapy-induced side effects, and improving *cancer patients’ quality of life*,^[16–18]^ especially in treating gastrointestinal adverse events,^[19]^ such as CINV,^[20,21]^ CID^[22]^ and CIC.^[23]^ Given that the underlying mechanism of chemotherapy-induced gastrointestinal adverse reaction remains unclear, previous studies suggested that it may correlated with multiple factors, including inflammation, secretory dysfunctions, dysmotility and innervation alterations in gastrointestinal.^[5]^ Previous studies have revealed that moxibustion is effective in alleviating inflammatory response, promoting immunity,^[24–26]^ regulating the gut microbiota and the membrane.^[27]^

However, current systematic reviews or meta-analyses did not focus on the efficacy of different types of moxibustion on different types of gastrointestinal adverse events induced by chemotherapy. This study will summarize the current evidences and conduct meta-analysis to evaluate the effectiveness and safety of moxibustion for patients who received chemotherapy and developed gastrointestinal adverse reaction. Moreover, it will help to define which gastrointestinal adverse symptom that moxibustion more focus on, to further provide instructions for clinical doctors, medical students and researchers.

## 2 Methods

### 2.1 Study registration

This protocol is reported in compliance with the preferred reporting items for systematic reviews and meta-analyses protocols (PRISMA-P) statement guidelines, and will be guided by the PRISMA guidelines and the recommendations of the Cochrane Handbook.^[28–30]^ The PROSPERO registration number is CRD42020182990.

### 2.2 Inclusion criteria for study selection

#### 2.2.1 Types of studies

All the RCTs of moxibustion for the management of chemotherapy-induced gastrointestinal adverse reactions will be included. Owing to the language restriction of the researchers, we will limit the language of search literature to Chinese and English. Non-RCTs, quasi-randomized trials, reviews, case reports, secondary analysis, animal experiments, or trials without full texts were excluded.

#### 2.2.2 Types of participants

We included participants diagnosed with cancer and suffered from gastrointestinal adverse reaction like nausea and vomiting, diarrhea and constipation after receiving chemotherapy. There will be no limits for age, gender, ethnic origin, and educational or economic status among patients. Patients with gastrointestinal adverse reaction caused by other reasons, will be excluded.

#### 2.2.3 Types of intervention

The intervention group should be treated by moxibustion, including direct moxibustion and indirect moxibustion. We excluded the trials that the intervention group is moxibustion combines with other conventional therapy or moxibustion is used as an adjuvant intervention, because it may difficult to evaluate the effectiveness of moxibustion alone. Basic chemotherapy treatment or supportive care should be identical in the intervention and control groups. Control group involving sham moxibustion, placebo, medication, no treatment will be included.

#### 2.2.4 Types of outcome measures

##### 2.2.4.1 Primary outcomes

The primary outcomes will be defined as the incidence and severity of chemotherapy-related gastrointestinal toxicities (CINV, CID, and CIC) by using validated scales to determined.

##### 2.2.4.2 Secondary outcomes

The European organization for research and treatment of cancer quality of life questionnaire-C30 (EORTC QLQ-C30) was used to assess quality of life. Changes in biological parameters aimed at assessing side effects of chemotherapy (such as inflammatory cytokine counts, gut microbiota or immunity response). Adverse events will also be assessed.

### 2.3 Data sources

Our systematic review will search all RCTs for moxibustion on chemotherapy-induced gastrointestinal adverse reaction, electronically and manually, regardless of publication status, till May 1, 2020. Online databases include PubMed, EMBASE, the Cochrane Library, Web of Science, China National Knowledge Infrastructure (CNKI), VIP Database and WanFang Database. Ongoing trials with unpublished data will be retrieved from the 4 following clinical trial registries: the Australian New Zealand Clinical Trials Registry (ANZCTR), the Clinical Trials.gov, World Health Organization (WHO) and the International Clinical Trials Registry Platform (ICTRP). The reference lists of the selected studies and published systematic reviews will be screened for additional studies. Manually search for the grey literature, including conference proceedings.

### 2.4 Search strategy

Two reviewers will independently search the studies in electronic databases according to the systematic review protocol. According to intervention, patients, and study type, the following search terms are used: moxibustion, chemotherapy, nausea and vomiting, CINV, emesis, diarrhea, CID, constipation, CIC, randomized, randomized controlled trial and so on. The search strategy will be adapted to different databases demands. Search strategy in PubMed is shown in Table 1.

**Table 1:**
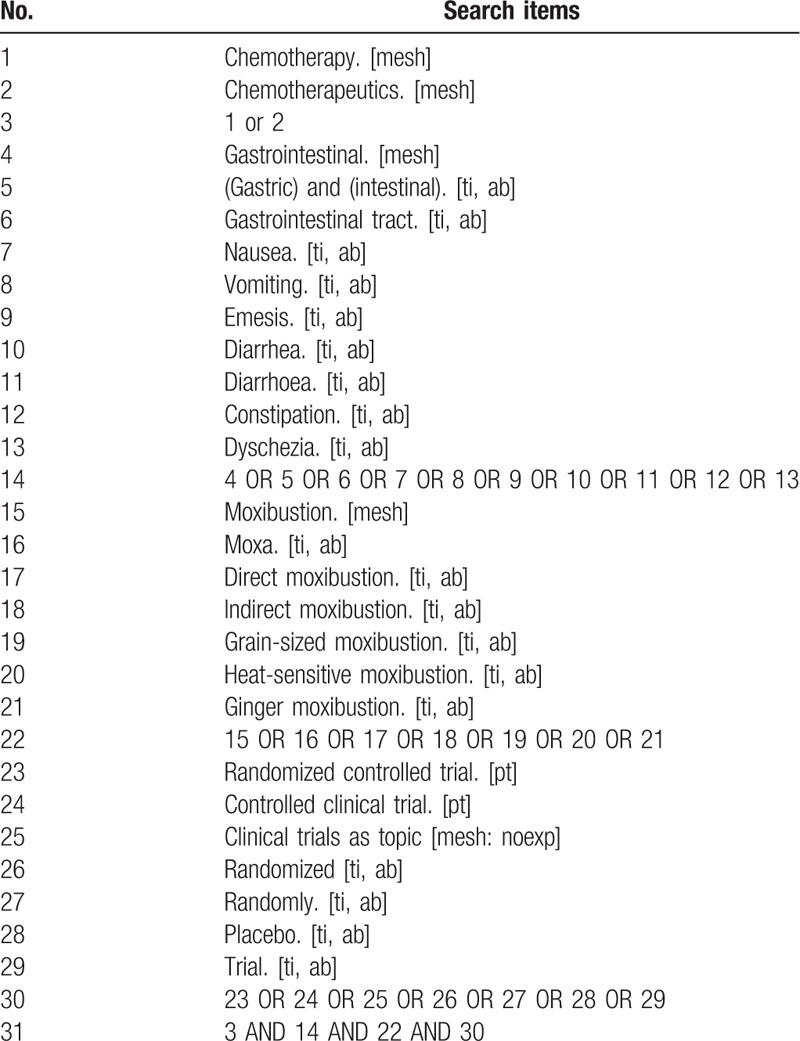
Search strategy in PubMed database.

### 2.5 Data collection and analysis

#### 2.5.1 Selection of studies

**Figure 1:**
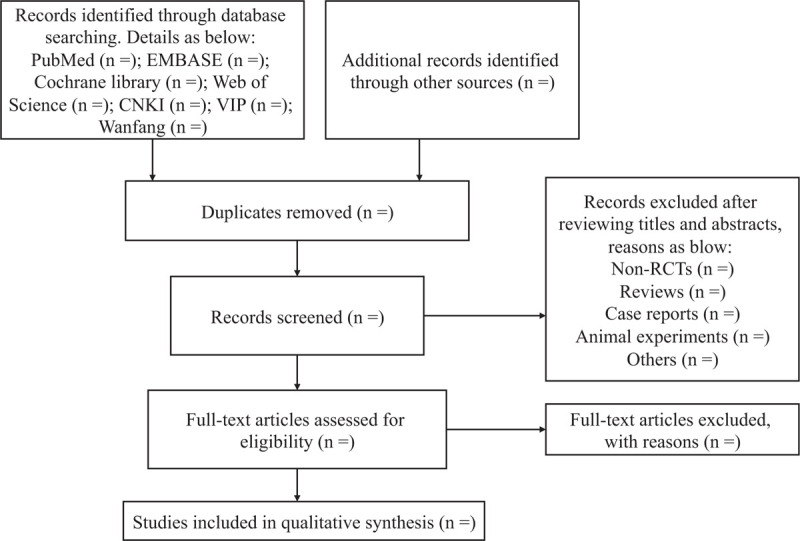
The preferred reporting items for systematic reviews and meta-analyses protocols (PRISMA-P) flow diagram of study selection process.

During the literature screening process, search results will be imported into Endnote X9 software and the duplicated studies will be deleted. The preliminary study selection will be independently performed by 2 researchers, who will screen all retrieval researches, read the titles, abstracts and keywords to determine which studies meet the inclusion criteria. We will obtain the full texts of all relevant studies for further evaluation. Studies excluded after reading the full text will be recorded and explained. The selection results will be cross-checked by the two researchers. Any disagreement between two researchers will be discussed and decided by a third researcher. The primary selection process is shown in Figure 1.

#### 2.5.2 Data extraction and management

The data will be extracted by 2 researchers independently using a predefined data extraction form. Data extracted from the included studies will cover the characteristics of study (authors, publication year, publication language, country, design, and setting), participants (age, gender, sample size, diagnostic and reason for moxibustion), intervention dose (duration, frequency and intensity), acupoints formula, comparisons, treatment outcomes, measurement tools, and adverse events. Disagreements will be resolved after discussed with a third researcher. For publications with insufficient or ambiguous data, we will attempt to obtain information from the corresponding author via e-mail or telephone.

#### 2.5.3 Assessment of risk of bias and reporting of study quality

Two researchers will independently evaluate the risk of bias using the Cochrane Collaboration's bias risk assessment tool. The following factors will be assessed: selection bias (random sequence generation and allocation concealment), performance bias (blinding of the investigators and participants, detection bias (blinding of the outcome assessment), attrition bias (incomplete outcome data), reporting bias, and possible other sources of bias. Disagreements between 2 researchers over the risk of bias in particular studies will be resolved by discussed with a third researcher. We assessed publication bias by using funnel plots.

#### 2.5.4 Measures of treatment effect

The results of the studies will be presented as risk ratio (RR) with 95% confidence interval (CI) for dichotomous data, whereas the continuous data will be presented as standardized mean difference (SMD) with 95% CI.

#### 2.5.5 Assessment of heterogeneity

The heterogeneity between studies were measured using the *I*^*2*^ statistic: when *I*^*2*^ is less than 50%, a fixed-effect model will be used for pooled data; otherwise, a random-effect model will be considered.

#### 2.5.6 Assessment of reporting biases

Funnel plots will be used to evaluate the publication biases if a sufficient number of included studies (more than 10 trials) are available.

#### 2.5.7 Data synthesis

The Cochrane Collaboration's Review Manager (RevMan 5.3) software will be used to conduct the direct meta-analysis. For dichotomous data, the RR with 95% CI was utilized for analysis. The fixed-effect model will be put into use if *I*^*2*^ < 50% or *I*^*2*^ > 75%, *I*^*2*^ < 50% will be classified as having low heterogeneity, whereas those with *I*^*2*^ > 75% will be classified as having high heterogeneity. When *I*^*2*^ > 75%, we will then perform a subgroup analysis or a sensitivity analysis.

#### 2.5.8 Subgroup analysis

Subgroup analyses will be used if there is an adequate number of studies, according to the type of moxibustion therapy (direct moxibustion, indirect moxibustion), the type of control group, the treatment duration, the clinical manifestation of chemotherapy-induced gastrointestinal adverse reaction like CINV, CID, and CIC.

#### 2.5.9 Sensitivity analysis

Multiple sensitivity analysis will be performed when sufficient trials are available, to assess the robustness of the summary estimates and to detect if any particular study accounted for a large proportion of heterogeneity. These will be based on different statistical approach, heterogeneity quality and sample size. The meta-analysis will be reused, and more inferior quality studies will be excluded.

#### 2.5.10 Grading the quality of evidence

We will use the Grading of Recommendations Assessment, Development and Evaluation (GRADE) to evaluate the quality of evidence which is classified into 4 levels: very low, low, moderate, or high.

## 3 Discussion

Gastrointestinal adverse reaction is one of the most common unavoidable harmful events during chemotherapy, may leading to treatment interruption and delay, even discontinuation among cancer patients.^[5]^ To date, given that the underlying mechanisms of chemotherapy-induced gastrointestinal adverse effects remain unclear, the current pharmacological interventions mainly take strategies on relieving symptoms, while did not receive satisfied results. Non-pharmacological therapies have shown their advantages, moxibustion, is one of the useful and safety methods, and widely used in Asia countries. Studies have reported that moxibustion is an effective therapy, that could reduce the inflammatory expression, regulate gut microbiotic and the gastrointestinal motility, to improve the QoL of cancer patients undergoing chemotherapy, besides, moxibustion has fewer side effects and relatively lower cost.

However, most of the current studies mainly focused on acupuncture rather than moxibustion, the recent published studies in 2020 are prepared to evaluate the CINV with acupuncture, which did not mention moxibustion.^[31,32]^ Most studies discuss the combination effects of other interventions and moxibustion,^[33]^ the effectiveness of moxibustion alone on chemotherapy-induced gastrointestinal adverse reaction should be evaluated. Although the previous published systematic review focus on moxibustion therapy alone on treating CINV, it was only included studies from 2005 to 2016,^[14]^ the included trials should be proper and updated.

Moxibustion has been identified to be effective in treating multiple types of chemotherapy-associated gastrointestinal adverse events, our study is aiming to critically evaluate all the RCTs of cancer patients receiving chemotherapy and displayed gastrointestinal adverse effects, and analyze moxibustion is inclined to treat which kind of gastrointestinal side symptoms. Besides, we will examine the effect and safety of different types of moxibustion on gastrointestinal adverse reaction, hoping to provide convincing evidence for clinicians when made decisions.

## Author contributions

**Conceptualization**: Han-Xiao Zhang

**Data curation**: Peng Lv, Chuan-Yi Zuo, Rui-Zhen Huang

**Formal analysis**: Qian-Ning Mo, Yi-Feng Bai

**Funding acquistion**: Cheng-Shun Zhang, Xiao-Qin Dai

**Investigation**: Xiao-Qin Dai

**Methodology**: Han-Xiao Zhang, Chuan-Yi Zuo

**Software**: Han-Xiao Zhang, Yi Zhou

**Supervision**: Cheng-Shun Zhang

**Writing – original draft**: Han-Xiao Zhang

**Writing – review & editing**: Han-Xiao Zhang, Cheng-Shun Zhang, Xiao-Qin Dai
